# Impaired Adipose Anabolism in Pancreatic Cancer Cachexia Is Reversed by HuR Inhibition

**DOI:** 10.1002/jcsm.70253

**Published:** 2026-03-19

**Authors:** Paige C. Arneson‐Wissink, Katherine Pelz, Beth Worley, Heike Mendez, Peter Pham, Parham Diba, Peter R. Levasseur, Grace McCarthy, Alex Chitsazan, Jonathan R. Brody, Aaron J. Grossberg

**Affiliations:** ^1^ Brenden‐Colson Center for Pancreatic Care Oregon Health & Science University Portland Oregon USA; ^2^ Department of Radiation Medicine Oregon Health & Science University Portland Oregon USA; ^3^ Department of Surgery Oregon Health & Science University Portland Oregon USA; ^4^ Cancer Early Detection Advanced Research Center Oregon Health & Science University Portland Oregon USA; ^5^ Department of Cell, Developmental, and Cancer Biology Oregon Health & Science University Portland Oregon USA

**Keywords:** adipogenesis, adipose, cachexia, HuR, pancreatic ductal adenocarcinoma

## Abstract

**Background:**

Cachexia is defined by chronic loss of fat and muscle, is a frequent complication of pancreatic ductal adenocarcinoma (PDAC) and negatively impacts patient outcomes. Nutritional supplementation cannot fully reverse tissue wasting, and the mechanisms underlying this phenotype are unclear. This work aims to define the relative contributions of catabolism and anabolism to adipose wasting in PDAC‐bearing mice. Human antigen R (HuR) is an RNA‐binding protein recently shown to suppress adipogenesis. We hypothesize that fat wasting results from a loss of adipose anabolism driven by increased HuR activity in adipocytes of PDAC‐bearing mice.

**Methods:**

Adult C57BL/6J mice received orthotopic PDAC cell (*Kras* ^*G12D*^; *p53* ^*R172H/+*^; *Pdx1‐cre*) (PDAC) or PBS (sham) injections. Mice exhibiting moderate cachexia (9 days after injection) were fasted for 24 h, or fasted 24 h and refed 24 h before euthanasia. A separate cohort of PDAC mice were treated with an established HuR inhibitor (KH‐3, 100 mg/kg) and subjected to the fast/refeed paradigm. We analysed body mass, gross fat pad mass and adipose tissue mRNA expression. We quantified lipolytic rate as the normalized quantity of glycerol released from 3T3‐L1 adipocytes in vitro and gonadal fat pads (gWAT) ex vivo.

**Results:**

3T3‐L1 adipocytes treated with PDAC cell conditioned media (CM) had lower expression of lipolysis and lipogenesis genes than control cells and did not display elevated lipolysis as measured by liberated glycerol. PDAC gWAT cultured ex vivo displayed decreased lipolysis compared to sham gWAT (−54.7%). PDAC and sham mice lost equivalent fat mass after a 24 h fast; however, PDAC mice could not restore inguinal fat pads (iWAT) (−40.5%) or gWAT (−31.8%) mass after refeeding. RNAseq revealed 572 differentially expressed genes in gWAT from PDAC compared to sham mice. Downregulated genes (*n* = 126) were associated with adipogenesis (adj *p* = 0.05), and expression of adipogenesis master regulators *Pparg* and *Cebpa* were reduced in gWAT from PDAC mice. Immunohistochemistry revealed increased HuR staining in gWAT (+74.9%) and iWAT (+41.2%) from PDAC mice. Inhibiting HuR binding restored lipogenesis in refed animals with a concomitant increase in iWAT mass (+131.7%).

**Conclusions:**

Our work highlights deficient adipose anabolism as a driver of reduced lipid content in 3T3‐L1 adipocytes treated with PDAC conditioned media and PDAC mice. The small molecule KH‐3, which disrupts HuR binding, restored adipose anabolism in PDAC mice. This highlights HuR as a potentially targetable regulatory node for adipose anabolism in cancer cachexia.

## 1 Introduction

Cancer‐associated cachexia is a wasting condition characterized by systemic inflammation, progressive weight loss, and atrophy of white adipose tissue (WAT) and skeletal muscle [1, S1, S2]. In addition to physical deterioration, individuals with cachexia also exhibit fatigue, anorexia and cognitive decline [1, S1–S3], which contribute significantly to reductions in quality of life, ability to tolerate chemotherapy or surgery and patient mortality [2, 3, S4–S6]. Cachexia is estimated to be the direct cause of death in 20%–30% of cancer patients [2, S4], and among all malignancies, pancreatic ductal adenocarcinoma (PDAC) is the most highly associated with cachexia, with an estimated 83% of patients suffering from this condition [4, S7, S8]. Despite much of cachexia research focusing on improving skeletal muscle mass, a retrospective study of patients with PDAC revealed that fat loss alone is associated with equally poor outcomes as combined muscle and fat mass loss [5]. Additionally, current cachexia clinical trials center around weight maintenance or gain as a primary endpoint. This highlights the importance of understanding the drivers of adipose tissue loss in cachexia.

Cachexia arises when energy catabolism exceeds anabolism, leading to unsustainable levels of fat mobilization and muscle depletion. Multiple factors are known to enhance catabolism, including decreased secretion of anabolic hormones and altered metabolism of protein, carbohydrate and lipid substrates [6]. Current work suggests that inflammation drives metabolic abnormalities in cachexia [7, S7]. Pro‐inflammatory cytokine activity increases during cancer progression [8, 9] and systemic inflammation can contribute to wasting by inducing hypercatabolism in muscle and adipose tissue [6, 10, 11, 12]. Existing literature highlights elevated rates of lipolysis and adipose browning as the primary forces underlying adipose tissue wasting [13, 14]. Browning, in particular, appears to exert a double effect in cachexia by both reducing lipid stores and increasing energy expenditure [14]. However, impaired anabolic processes, like adipogenesis and lipogenesis, also contribute to adipose tissue loss [15]. Targeting peroxisome proliferator–activated receptor gamma (PPARG), a transcriptional control point of adipogenesis, with the agonist rosiglitazone was sufficient to improve fat and muscle mass retention in mice with lung cancer [16]. Tumour‐derived factors are also capable of impairing adipogenesis in cultured 3T3L‐1 adipocytes [17, 18].

RNA‐binding proteins (RBPs) are essential in governing biogenesis, stabilization, translation and decay of mRNA transcripts [19, 20]. In adipose tissue, several RBPs are documented to regulate alternative splicing [21, 22, 23], control of key adipogenic transcription factors [24] and the translation efficiency of proteins involved in browning [25]. Until recently, the function of most RBPs in adipocytes was largely unexplored. The RBP human antigen R (HuR), encoded by the embryonic lethal abnormal vision‐like 1 (*ELAVL1*) gene, regulates the expression of genes involved in inflammation, stress response and apoptosis [26]. Although HuR likely exerts complex effects in the context of both PDAC and cachexia, we selected HuR as a target of interest because recent work showed that it is a repressor of adipogenesis in both white and brown adipose tissues [27]. Our goal was to elucidate the relative contributions of enhanced catabolism and impaired anabolism on fat wasting by investigating adipose tissue response to different nutritional contexts and HuR inhibition in cachectic mice.

## 2 Methods

### 2.1 Cell Culture

#### 2.1.1 KPC PDAC Cells

KPC cells expressing pancreas‐specific conditional alleles (*Kras* ^*G12D*^; *p53* ^*R172H/+*^; *Pdx1‐cre*) [28] were stored in liquid nitrogen until use and then maintained in RMPI 1640 supplemented with 10% FBS, 1 mM sodium pyruvate and 50 U/mL penicillin/streptomycin (Gibco, Gaithersburg, MD) at 37C and 5% CO_2_. Conditioned media (CM) were collected from confluent KPC cells grown in DMEM, 1% pen/strep (to accommodate later culturing of 3T3‐L1 cells) after 24 h incubation, centrifuged at 1200 g for 10 min, filtered with a 0.2‐μm syringe filter and used immediately or stored at −80°C.

#### 2.1.2 3T3‐L1 Adipocytes

3T3‐L1 adipocytes (ATCC CL‐173) were purchased from ATCC and stored in liquid nitrogen until use. Cells were cultured in preadipocyte expansion media (DMEM, 10% bovine calf serum, 1% pen/strep). Then, cells were plated for differentiation at a density of 2 × 10^5^ cells/six‐well dish, or 6.7 × 10^3^ cells/96‐well dish and maintained in preadipocyte expansion media for 2 days after reaching 100% confluency. Media were changed to adipocyte differentiation media (DMEM, 10% FBS, 1% pen/strep, 1 μM dexamethasone, 0.5 mM IBMX, 1 μg/mL bovine insulin) for 2 days before switching to adipocyte maintenance media (DMEM, 10% FBS, 1% pen/strep, 1 μg/mL bovine insulin) for the duration of the experiment. Cells were cultured for up to 6 days after start of maintenance media to reach complete differentiation. For Oil Red O staining, CM was added in place of adipocyte maintenance media and was supplemented with FBS and insulin for 6 days. For lipolysis assay (media glycerol, qPCR and western blot), cells were completely differentiated and then changed to control media, KPC conditioned media without insulin or FBS. Isoproterenol (10 μM) was added to media as a positive control. Lipolysis endpoints were collected at 1, 3 and 24 h after media change.

### 2.2 Oil Red O Staining

Diluted Oil Red O stock solution was prepared by mixing concentrated Oil Red O (0.5% Oil Red O in 100% isopropyl alcohol) 6:4 with deionized water. This solution was allowed to stand for 10 min at 4°C and then filtered immediately before use through a 0.22‐μm filter. 3 T3‐L1 cells were washed with PBS, then incubated in 4% paraformaldehyde for 30 min at room temperature and then washed with deionized water twice. Cells were incubated in 60% isopropyl alcohol for 5 min and then treated with diluted Oil Red O solution for 15 min. Cells were washed five times with deionized water, and then the red signal was quantified at 490 nm in a plate reader (BioTek).

### 2.3 Glycerol Quantification

3T3‐L1 adipocyte media were collected and run at a 1:2 dilution, and mouse plasma samples were run undiluted for glycerol quantification according to the manufacturer's instructions (Abcam ab65337). Fresh media and KPC CM alone (no 3T3‐L1 exposure) were below the detection limit of the assay.

### 2.4 NEFA Quantification

Mouse plasma was run undiluted according to the manufacturer's instructions (Wako Diagnostics NEFA‐HR series). mEq/L are the units specified by the assay manufacturer and what is standard reporting in clinical values. mEq/L = mM × |valence charge|. Because NEFAs have a valence charge of −1, mEq/L is equivalent to mM.

### 2.5 Mouse Studies

Wild‐type C57BL/6J mice (JAX catalogue number 000664) were purchased from The Jackson Laboratory (Bar Harbor, ME) and maintained in standard rodent housing at 26°C with 12‐h light/12‐h dark cycles. Sex is defined in individual figure legends. Animals used for experimentation were 12–15 weeks of age. Mice were individually housed for acclimation for 7 days prior to tumour implantation and provided *ad libitum* access to water and food (Rodent diet 5001; Purina Mills, St. Louis, MO, USA). Food intake was measured daily. Tumour‐bearing animals were euthanized by cardiac puncture under deep isoflurane anaesthesia. Studies were conducted in accordance with the US National Institutes of Health Guide for the Care and Use of Laboratory Animals and approved by the Institutional Animal Care and Use Committee of Oregon Health & Science University.

#### 2.5.1 Study‐Specific Manipulations

For fasting studies, mice were transferred to clean cages without food for 24 h prior to euthanasia or refeeding. Pair‐feeding was used on the study days indicated in the figure legends by feeding sham mice the average of the food consumed by PDAC mice the day prior. PDAC mice treated with the HuR inhibitor KH‐3 were injected intraperitoneally (100 mg/kg) at 6‐, 8‐ and 10‐day post‐implantation.

#### 2.5.2 Orthotopic PDAC Implantation

Wild‐type C57BL/6J mice aged 12–15 weeks received orthotopic PDAC tumour injections (*Kras* ^*G12D*^; *p53* ^*R172H/+*^; *Pdx1‐cre*) or sham injections. PDAC mice were injected with 1 × 10^6^ KPC cells into the tail of the pancreas parenchyma in a volume of 23 μL, whereas sham animals were treated with an equal volume of PBS. Animals were euthanized 10–11 days after tumour implantation. N and sex are defined on a per‐study basis in the figure legends.

#### 2.5.3 Echo Magnetic Resonance Imaging Body Composition

Lean mass, fat mass, total body water and free water were measured using whole‐body magnetic resonance imaging (MRI) (EchoMRI, Houston, TX). Measurements were taken pre‐implantation (baseline), pre‐fasting and at euthanasia in tumour and sham groups to assess body composition.

### 2.6 Tissue Collection and Histology

Tissues collected at necropsy were weighed and flash frozen in liquid nitrogen prior to storage at −80°C. Tissues for HuR histology were fixed with 4% paraformaldehyde overnight and then transferred to 70% ethanol. Tissues were paraffin‐embedded, sectioned, incubated with anti‐HuR (1:300 #sc‐5261, Santa Cruz) and stained using horseradish peroxidase–conjugated secondary antibody and incubation in 3,3′‐diaminobenzidine by the Histopathology Shared Resource Core at OHSU. Whole tissue sections were scanned by the Advanced Light Microscopy Core at OHSU. ZEN Digital Imaging for Light Microscopy (RRID:SCR_013672) was then used to obtain at least five 10× images per tissue. ImageJ software was used to quantify the percent staining for each section (56). All staining was quantified by running colour deconvolution on 10× images, applying a standard intensity threshold on the corresponding images and measuring the percent area covered by the staining.

### 2.7 Ex Vivo Lipolysis

Ex vivo lipolysis assays of adipose explants were performed as previously described [29]. Briefly, gonadal white adipose tissue (gWAT) tissue was collected from ad‐lib fed sham and PDAC mice 10 days after tumour implantation. Tissues were cut into approximately 100 mg samples, minced and then incubated in phenol red‐free DMEM containing 2% fatty acid free bovine serum albumin at 37°C for 1 h. Media were collected and snap‐frozen. The tissue was transferred to a new dish containing media only or media with 10 μM isoproterenol and incubated for 2 h at 37°C. Media were collected and snap‐frozen. Media aliquots were thawed and analysed for glycerol using Sigma Glycerol Assay Kit (MAK117) according to manufacturer's directions.

### 2.8 Quantitative qRT‐PCR

Total RNA was extracted from cell pellets or tissues with the E.Z.N.A. Total RNA Kit II (Omega Bio‐Tek Inc., Norcross, GA). cDNA was transcribed with the High‐Capacity cDNA Reverse Transcription Kit (Applied Biosystems, Waltham, MA). Quantitative real‐time polymerase chain reaction (qPCR) was run on the ABI 7300 (Applied Biosystems) using TaqMan Fast Advanced PCR Master Mix (Applied Biosystems) or SYBR Green Master Mix (Applied Biosystems). The relative expression was calculated using the ΔΔC_t_ method with gene expression relative to beta actin or 18S.

### 2.9 RNA Sequencing

RNA sequencing was performed on total RNA isolated as described above. RNA libraries were prepared and sequenced using the Illumina Nova Seq and HiSeq platform according to the Illumina Tru‐Seq protocol (Novogene, Sacramento, CA). Detailed analysis methods are included in the Supporting Information.

### 2.10 Western Blot

3T3‐L1 protein was collected by scraping cells into lysis buffer followed by brief sonication. Adipose tissue protein was collected using the Minute Total Protein Extraction Kit (Invent Biotechnologies Inc., AT‐022) and the manufacturer's protocol. We used BCA assay to determine protein concentration. Approximately 20 μg of protein was loaded in each lane and run on 4%–12% Bis‐Tris NuPAGE gel (Invitrogen). Gels were transferred to polyvinylidene difluoride (PVDF) membranes (Millipore) and blocked with 5% BSA for 1 h. Membranes were incubated with primary antibodies overnight at 4°C with gentle agitation. Blots were then washed with Tris‐buffered saline with Tween 20 (TBST) and incubated in secondary antibodies for 1 h prior to imaging (LI‐COR Odyssey Imaging System). Ladders used were iBright Pre‐stained Protein Ladder (Invitrogen) (HuR blot) and Chameleon 700 Pre‐stained Protein Ladder (LI‐COR) (all other blots). Primary antibodies: Vinculin (Santa Cruz sc‐73 614), HuR (Santa Cruz sc‐5261), ATGL (Cell Signaling 2138), HSL (Cell Signaling 4107), Phospho‐HSL (Ser660) (Cell Signaling 45804), and Phospho‐HSL (Ser563) (Cell Signaling 4139). Secondary antibodies: Mouse IgG and Rabbit IgG Dylight 680 and 800 (Cell Signaling), goat anti‐mouse IgG Alexa Fluor Plus 800 (Invitrogen A32730) and goat anti‐mouse IgG Alexa Fluor Plus 647 (Invitrogen A32728).

### 2.11 Statistical Analysis

Specific statistical tests and sample size for each study are indicated in the figure legends. Error bars in figures show SEM. Statistical analyses were performed using GraphPad Prism (Version 9; GraphPad Software Inc.), and graphs were built using GraphPad Prism (GraphPad Software Inc.) statistical analysis software or R Studio (4.2.3) using ggplot (3.5.1). The *p* values are two sided with values less than 0.05 regarded as statistically significant.

### 2.12 Availability of Data

Further information and resources, including KPC cells and raw data will be shared upon reasonable request to Aaron J. Grossberg (grossber@ohsu.edu).

## 3 Results

### 3.1 Adipocytes Suppress Lipogenesis in Response to PDAC‐Derived Factors

**FIGURE 1:**
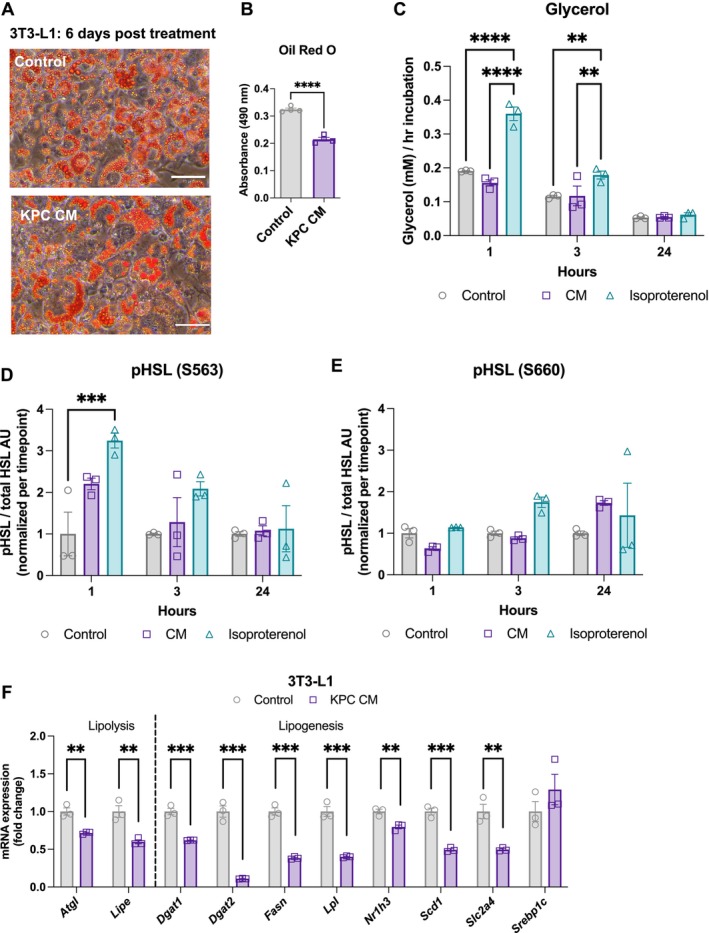
Adipocytes suppress lipogenesis in response to pancreatic ductal adenocarcinoma‐conditioned media. (A) Representative bright‐field images of differentiated 3T3‐L1 cells treated with control media (top) or KPC CM (bottom) for 6 days. Scale bars represent 70 μm. (B) Quantification of Oil Red O staining. *N* = 4 wells per condition. (C–E) 3T3‐L1 cells were fully differentiated and then treated with control, KPC‐conditioned or isoproterenol (10 μM) media for 1, 3 and 24 h in the absence of FBS and insulin. *N* = 3 wells per timepoint/condition. (C) Media glycerol levels, normalized by hours of collection. Conditioned media and control media alone did not contain glycerol above background (assay buffer only) levels. (D) Serine 563 phosphorylated HSL protein levels normalized to total HSL protein levels. (E) Serine 660 phosphorylated HSL protein levels normalized to total HSL protein levels. (F) mRNA expression of lipolysis and lipogenesis genes. *N* = 3 wells per condition, normalized to 18S expression. Panels (B) and (F) tested with *t*‐test. Panels (C–E) tested with two‐way ANOVA with Tukey correction for multiple comparisons. *p* < 0.05, **p < 0.01, ***p < 0.001, ****p < 0.0001.

Given a large body of literature suggesting that PDAC cachexia is partly driven by increased lipolysis in the adipose tissue, we tested the effect of PDAC cell conditioned media (KPC CM) on lipolysis in vitro [13, 14, 30]. We treated differentiated 3T3‐L1 cells with control maintenance media or with KPC CM for 6 days. At this time, we observed decreased lipid droplet accumulation by bright‐field microscopy, which was confirmed with Oil Red O (Figure 1A,B). Media glycerol levels, a measure of lipolysis, were not elevated in KPC CM‐treated cells (Figures 1C and S1A). Correspondingly, protein markers of lipolysis, including activated (phosphorylated) hormone‐sensitive lipase (HSL) and adipose triglyceride lipase (ATGL), were not elevated in KPC CM‐treated cells (Figures 1D,E and S1B–E and S2), and mRNA levels of lipolysis enzymes *Atgl* and hormone‐sensitive lipase (lipase e, *Lipe*) were significantly decreased in KPC CM‐treated 3T3‐L1 cells (Figure 1F). To explain the apparent decrease in lipid accumulation without increased lipolysis, we measured the expression of genes associated with lipogenesis: diacylglycerol O‐acyltransferase 1 and 2 (*Dgat1 and Dgat 2)*, fatty acid synthase (*Fasn*), lipoprotein lipase (*Lpl*), nuclear receptor subfamily 1 group H member 3 (*Nr1h3*), stearoyl‐CoA Desaturase 1 (*Scd1*), solute carrier family 2 member 4 (*Slc2a4*) and sterol regulatory element‐binding protein 1c (*Srebp1c*). All of these genes were suppressed after 24‐h KPC CM treatment, indicating that the decreased lipid content in 3T3‐L1 cells was due to impaired anabolic activity rather than increased catabolic activity (Figure 1F).

### 3.2 PDAC Is Associated With Decreased Fat Pad Mass In Vivo

**FIGURE 2:**
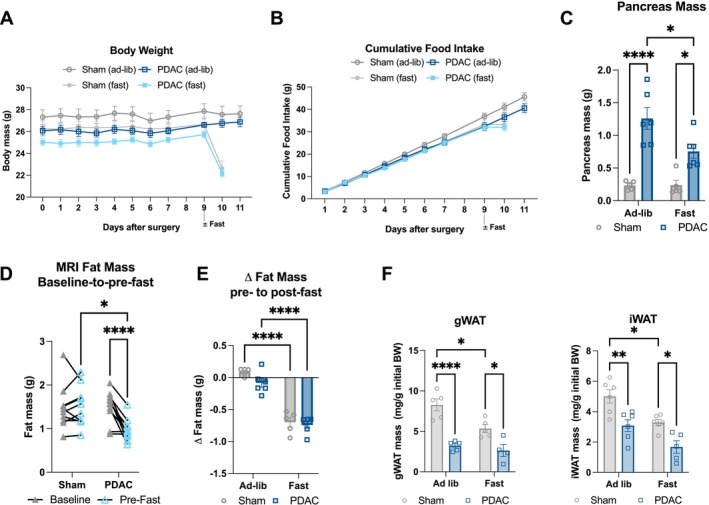
Pancreatic ductal adenocarcinoma is associated with decreased fat pad mass in vivo. (A–E) Wild‐type C57BL/6J mice with PDAC or sham implantations were fed *ad libitum* or fasted 16 h at mid‐cachexia (9 days after injection). Animals were euthanized 10 or 11 days after tumour implantation following a 16‐h fast. *N* = 5 sham, 6 PDAC male mice per feeding condition. (A) Daily body mass. Statistically tested with three‐way ANOVA *p* < 0.0001 for time, time × fast; *p* = 0.0073 for fast; *p* = 0.0097 for time × tumour status; *p* = 0.0167 for tumour status. (B) Cumulative food intake. Statistically tested with three‐way ANOVA *p* < 0.0001 for time, time × fast; *p* = 0.0035 for time × tumour status. (C) Terminal pancreas/tumour mass. (D,E) Body composition changes in total adiposity were characterized from baseline (pre‐tumour implantation) to pre‐fast between sham and PDAC mice (C) and from pre‐ to post‐fast (D). (F) Terminal gWAT and iWAT mass following 24‐h fast 14 days after injection. *N* = 5 male mice per ad lib group, 5 male mice sham fast, 3 male mice PDAC fast. 2 × 2 analyses were statistically tested with two‐way ANOVA or mixed effects model with Tukey multiple comparisons. *p < 0.05, **p < 0.01, ****p < 0.0001.

We first sought to assess the effects of pancreatic cancer on metabolism by characterizing tissue physiology in a murine orthotopic PDAC model. Twelve‐week‐old C57BL/6J mice with PDAC or sham implantations were fed *ad libitum* with or without a 24‐h fast once mice reached a moderate cachexia burden (9 days after tumour implantation). Body mass maintained stable among all groups prior to fasting (Figure 2A), whereas PDAC mice exhibited a small reduction in food intake (Figure 2B) compared to sham controls. Fasting caused a significant reduction in tumour mass compared to *ad libitum* fed mice (Figure 2C). EchoMRI body composition analysis of fat mass demonstrated significant decreases in overall adiposity in PDAC animals from baseline to pre‐fast due to cachexia progression and reduction in food intake (Figure 2D). As expected, between pre‐ and post‐fast, both PDAC and sham animals lost significant overall adiposity (Figure 2E). Terminal fat pad masses (inguinal, iWAT and gonadal, gWAT) were significantly decreased in both ad lib and fasted conditions as compared to sham mice (Figure 2F). PDAC mice were also vulnerable to skeletal muscle mass loss as measured by EchoMRI and gastrocnemius muscle tissue mass (Figure S3).

### 3.3 PDAC Impairs Lipolysis and Adipogenesis

**FIGURE 3:**
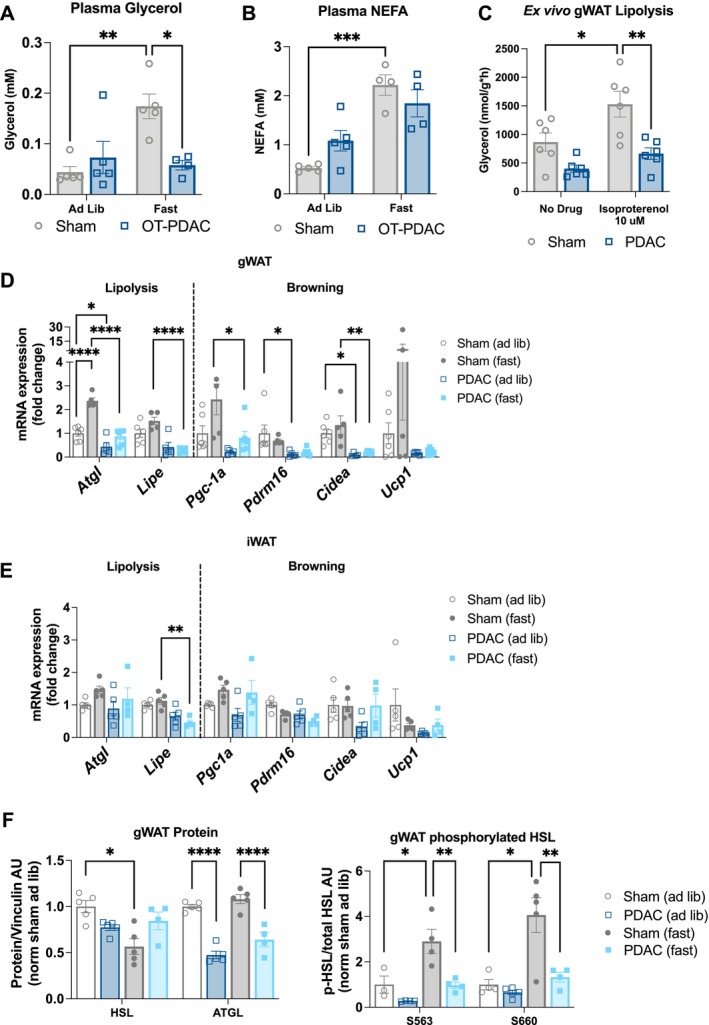
Pancreatic ductal adenocarcinoma impairs lipolysis and adipogenesis in vivo. (A) Measurement of lipolysis (glycerol release) in explants derived from gWAT collected 10 days after tumour implantation. Lipolysis was induced by the β‐adrenergic agonist isoproterenol (10 μM). *N* = 6 male mice per group. Statistically tested with two‐way ANOVA with uncorrected Fisher's LSD test to compare groups. (D) mRNA levels of lipolysis and browning genes in gWAT collected 10 days post‐implantation from 24‐h‐fasted and *ad libitum* fed mice, normalized to 18S expression. *N* = 5 male mice sham/fasted; 3 male, 3 female mice sham/ad lib; 6 male mice PDAC/fasted; 3 male, 2 female mice PDAC/ad lib. (E) mRNA expression of lipolysis and browning genes in iWAT collected 11 days post‐implantation from *ad libitum* fed mice, normalized to beta actin expression. *N* = 4 male mice per group. (F) Western blot analysis of PDAC and sham gWAT protein for HSL (83 kDa) and ATGL (55 kDa) (left) and serine 563‐ and serine 660‐phosphorylated HSL protein levels normalized to total HSL protein levels (right). *N* = 5 male mice per group. Protein normalized to vinculin (124 kDa). 2 × 2 data were statistically tested with two‐way ANOVA with Tukey multiple comparisons comparing tumour experimental groups to sham controls. Pairwise comparisons were made with unpaired *t*‐test. *p < 0.05, **p < 0.01, ***p < 0.001, ****p < 0.0001.

Based on our findings in vitro, we hypothesized that PDAC mice would not exhibit increased lipolysis, which is characteristic of other cachexia models [13, 30]. To assess products of lipolysis in circulation, we measured plasma glycerol and non‐esterified fatty acids (NEFAs). Plasma glycerol levels were significantly lower in fasted PDAC mice than in fasted sham mice (Figure 3A), although there was no significant difference in plasma NEFA levels between fasted groups (Figure 3B). To directly measure the rate of adipose tissue catabolism, we performed an ex vivo lipolysis study, using gWAT collected from sham and PDAC mice. We assessed fat pads both in the presence and absence of the beta‐adrenergic agonist isoproterenol (10 μM) to determine baseline and stimulated lipolysis [29]. Baseline and isoproterenol‐stimulated glycerol release were significantly decreased in PDAC gWAT (Figure 3C), demonstrating that PDAC suppresses, rather than enhances, lipolytic rate and capacity. To further characterize adipose tissue in PDAC mice, we assessed mRNA expression of genes regulating lipolysis and browning in adipose tissue. PDAC mice had suppressed protein and mRNA expression of ATGL and suppressed activation (phosphorylation) of HSL in the gWAT (Figures 3D,F and S4). PDAC mice also showed suppressed iWAT and gWAT *Lipe* expression under fasted conditions (Figure 3D,E). We observed suppressed browning‐associated gene expression in gWAT, including the genes peroxisome proliferator–activated receptor gamma coactivator 1 alpha (*Pgc1a*), protein domain containing 16 (*Prdm16*) and cell death inducing DFFA like effector a (*Cidea*) in PDAC mice in both *ad libitum* fed and fasted conditions. Uncoupling protein 1 (*Ucp1*), which is also associated with browning, was not significantly increased in gWAT or iWAT from PDAC mice (Figure 3D,E). Therefore, neither enhanced lipolysis nor browning is a significant contributor to adipose loss in this model.

### 3.4 PDAC Downregulates Pathways Associated With Adipogenesis

**FIGURE 4:**
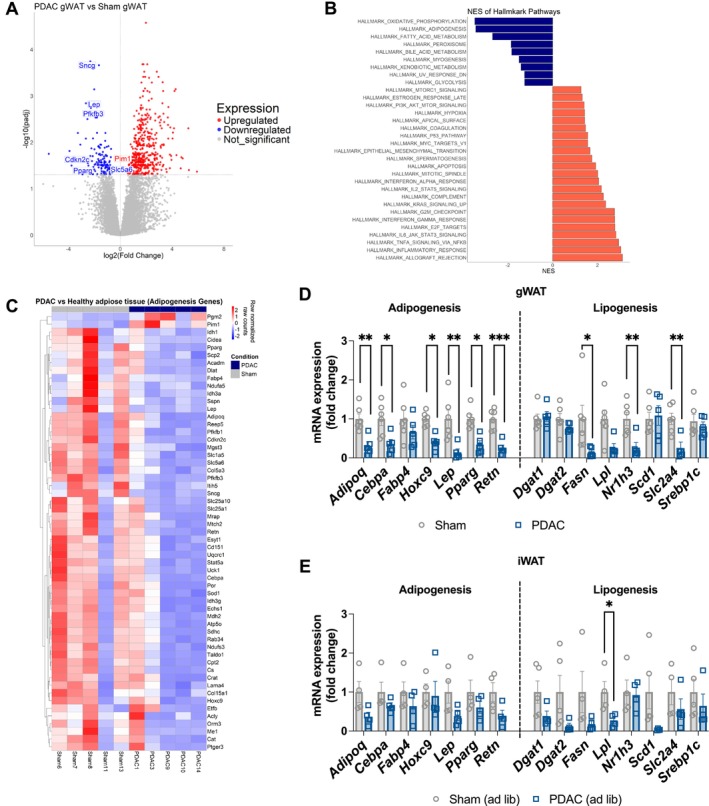
PDAC downregulates pathways associated with adipogenesis. RNAseq on gWAT from *ad libitum* fed PDAC and sham mice collected 14 days post tumour implantation. *N* = 2 female, 3 male PDAC; 3 female, 2 male sham. (A) Volcano plot of differentially expressed genes in gWAT. Blue dots represent significantly downregulated genes (log_2_ fold change < −1 and adj *p* < 0.05). Red dots represent significantly upregulated genes (log_2_ fold change > 1 and adj *p* < 0.05). Grey dots are insignificant. Labels denote significant differentially expressed genes from the Hallmark Adipogenesis pathway. (B) Broad Institute GSEA showing significantly enriched pathways in PDAC gWAT relative to sham gWAT (pathway *p* < 0.05). Blue is decreased expression, and red is increased expression. (C) Heat map of Hallmark Adipogenesis pathways constituents in PDAC and sham gWAT. Scale represents row‐normalized raw counts. (D) qPCR validation of adipogenesis and lipogenesis genes in gWAT collected 10 days post‐implantation from 24‐h‐fasted and *ad libitum* fed mice. *N* = 5 male mice sham/fasted; 3 male, 3 female mice sham/ad lib; 6 male mice PDAC/fasted; 3 male, 2 female mice PDAC/ad lib. Statistically tested with two‐way ANOVA with Tukey multiple comparisons comparing tumour experimental groups to sham controls. (E) qPCR validation of adipogenesis and lipogenesis genes in iWAT from ad lib fed mice collected 11 days post implantation. *N* = 4 male mice per group. Statistically tested with unpaired *t*‐test. *p < 0.05, **p < 0.01, ***p < 0.001, ****p < 0.0001.

To gain further insights into PDAC metabolism from global gene expression analysis, we performed bulk RNA sequencing (RNAseq) on gWAT from sham and PDAC animals. In gWAT, we identified a total of 572 differentially expressed genes (DEGs) in PDAC versus sham mice, of which 446 were enriched and 126 were depleted (Figures 4A and S5). Pathway analysis referencing the molecular signatures database (MSigDB) hallmark gene set collection revealed increased expression of gene sets associated with cell cycle control (e.g., E2F targets and G2M checkpoints) and inflammation (e.g., TNFα and JAK/STAT3 signalling) and decreased expression of genes linked to adipogenesis, oxidative phosphorylation and fatty acid metabolism (Figure 4B) [31]. Because inhibition of adipogenesis could provide an alternative mechanism for adipose wasting, we plotted all significant DEGs in the adipogenesis gene set and observed nearly universal depletion of these transcripts in PDAC mice (Figure 4C). We then validated our RNAseq data by performing qPCR on selected adipogenesis‐associated DEGs, revealing consistent downregulation of both adipogenesis and lipogenesis genes (Figure 4D). We then repeated qPCR in iWAT to determine whether subcutaneous adipose exhibited the same transcriptomic changes. Transcriptional changes in iWAT were less dramatic than in gWAT and did not reach significance, with the exception of *Lpl*, which was significantly decreased in PDAC tissue (Figure 4E). Together, these results indicate that suppressed transcriptomic programmes associated with adipogenesis could account for decreased adipose tissue during cachexia in the absence of elevated lipolysis.

### 3.5 Adipose Tissue Anabolism Is Impaired in Orthotopic PDAC Mice After Refeeding

**FIGURE 5:**
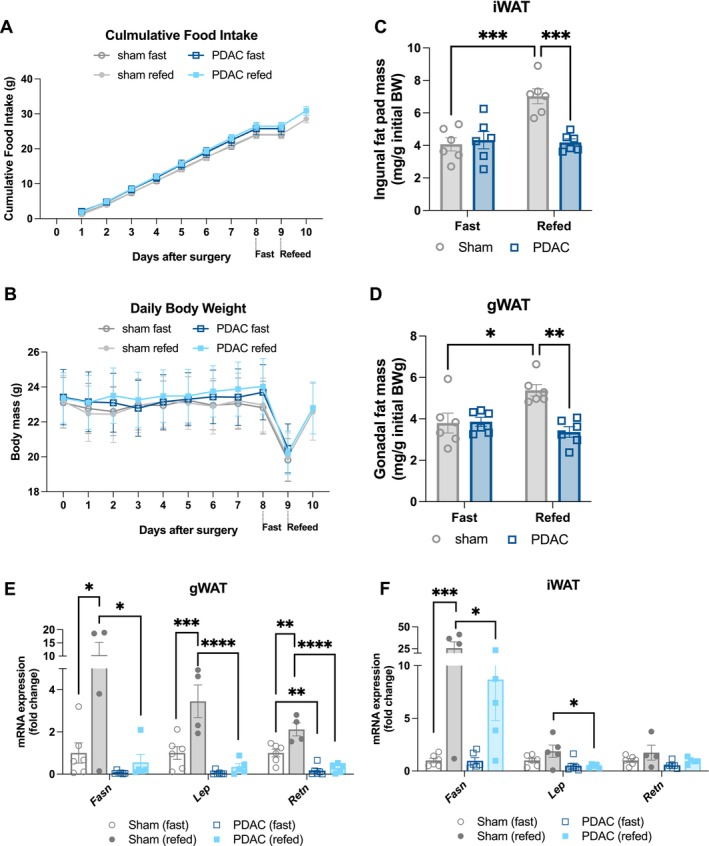
Adipose tissue anabolism is impaired in orthotopic PDAC mice after refeeding. Wild‐type C57BL/6J mice with PDAC were fed *ad libitum* for 9 days and then fasted 24 h with or without a 24‐h refeeding period. Sham mice were pair‐fed daily to the average food intake of the PDAC group from 2 days post‐implantation until study end. Animals were euthanized 10 or 11 days after tumour implantation following the 24‐h fast (10 days) or 24‐h refeed (11 days). *N* = 3 male and 3 female mice per group. (A) Cumulative food intake. Statistically tested with three‐way ANOVA. *p* < 0.0001 for time, *p* = 0.0344 for time × tumour status. (B) Daily body mass. Statistically tested with three‐way ANOVA. *p* < 0.0001 for time, *p* = 0.002 for time × tumour status. iWAT (C) and gWAT (D) mass normalized to initial body mass. mRNA expression of anabolic genes in gWAT (E) and iWAT (F), normalized to beta actin expression. Data represented in (C–F) were statistically tested with two‐way ANOVA with Tukey multiple comparisons comparing PDAC groups to sham controls. *p < 0.05, ****p < 0.0001.

Based on our results demonstrating downregulation of adipogenic and lipogenic genes in PDAC mice, we next wanted to confirm whether adipose tissue anabolism is functionally impaired in mice implanted with orthotopic PDAC. To do this, wild‐type C57BL/6J mice with PDAC or sham implantations were fed *ad libitum* for 9 days, an established timepoint of active cachexia [32], fasted 24 h and then terminated or allowed to refeed for 24 h. To control for differences in caloric intake, we used a pair‐fed refeeding scheme in which sham mice were refed with the average food consumption of the refed PDAC group. Challenging mice with a 24‐h fast depletes adipose mass in both PDAC and sham mice (Figure 2D), enabling us to assess anabolic restoration of fat mass during a 24‐h refeeding period. We measured cumulative food intake and daily body mass for the duration of the study (Figure 5A,B). A 24‐h fasting followed by 24‐h refeeding did not impact pancreas tumour mass (Figure S6). There were no differences in iWAT or gWAT mass in fasted PDAC versus sham mice (Figure 5C,D). However, although sham mice regain significant amounts of gWAT and iWAT mass after refeeding, gWAT and iWAT masses in PDAC mice remain equivalent before and after refeeding (Figure 5C,D). Refeeding sham mice caused increased gWAT expression of lipogenic *Fasn* and adipogenic genes *Lep* and *Retn*, although these genes remained suppressed in PDAC gWAT (Figure 5E). Refed sham iWAT showed increased *Fasn*, but not adipogenic genes, whereas PDAC iWAT had suppressed expression of both adipogenic and lipogenic genes (Figure 5F). These results confirm that downregulation in pathways mediating adipogenesis is indeed accompanied by impaired adipose tissue anabolism in orthotopic PDAC mice after refeeding.

### 3.6 HuR Is Highly Expressed in Orthotopic PDAC Fat Tissue

**FIGURE 6:**
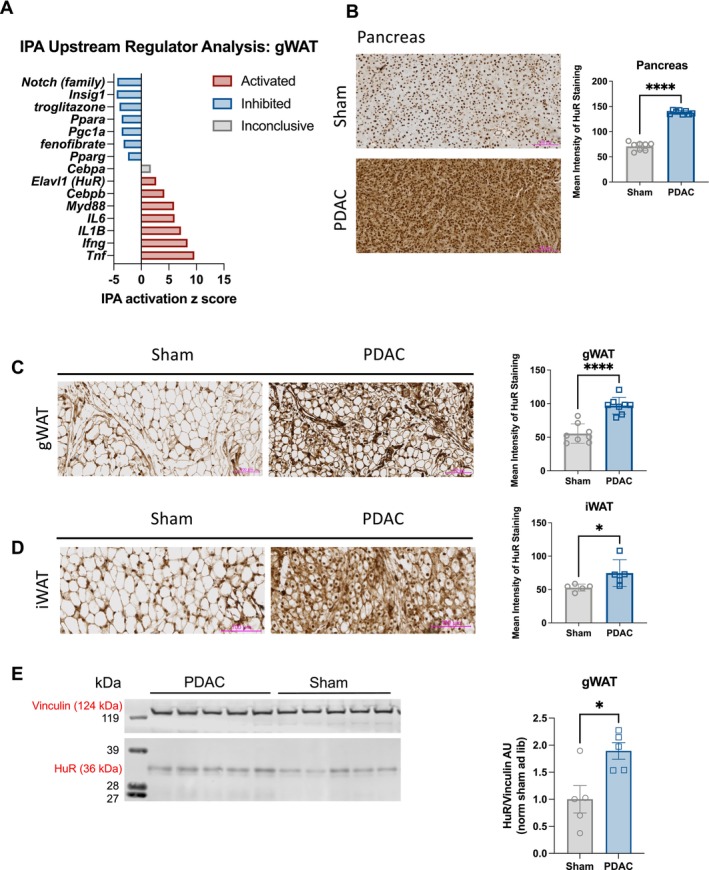
HuR is highly expressed in PDAC orthotopic pancreas and fat tissue. (A) Selected upstream regulator predictions from Ingenuity Pathway Analysis of differentially expressed genes in gWAT. (B–D) Representative images and quantification of immunohistochemical detection of HuR in tissue from PDAC and sham mice. (B) Pancreas *N* = 4 male and 4 female mice per group. (C) gWAT *N* = 4 male and 4 female mice per group. (D) iWAT *N* = 3 female, 2 male sham and 2 female, 3 male PDAC. (E) Western blot analysis of PDAC and sham gWAT protein for HuR. *N* = 5 male mice per group. HuR protein (36 kDa) normalized to vinculin protein (124 kDa). Scale bars represent 100 um. Statistically tested with *t*‐test. *p < 0.05, **p < 0.01, ***p < 0.001, and ****p < 0.0001.

Following our observation that adipose tissue anabolism is impaired in PDAC animals, we next sought to understand the mechanism of this phenomenon. We used Ingenuity Pathway Analysis upstream regulator analysis to identify potential candidates that could drive impaired anabolism in PDAC adipose tissue. From the DEGs identified in gWAT of PDAC mice, we found that canonical regulators of adipogenesis, such as troglitazone, fenofibrate, PPARA and PGC1A, were predicted to be inhibited. Inflammatory cytokines associated with PDAC and cachexia, such as MYD88, IL‐6, TNFA, IFNG and IL‐1B, were predicted to be activated. Among other targets that were predicted to be activated but were not typically associated with cachexia was HuR (*ELAVL1*), an RBP recently established as a suppressor of adipogenesis [27] (Figure 6A). Given the predicted activation of HuR in gWAT and existing literature, we next asked if HuR was more abundant in adipose tissue from PDAC mice. We performed immunohistochemical staining of HuR in formalin‐fixed, paraffin‐embedded sham and PDAC gWAT, iWAT, pancreas and muscle tissue. HuR staining was significantly increased in the pancreas, gWAT and iWAT of PDAC mice versus sham controls (Figures 6B–D and S7–S8). We also observed increased HuR protein abundance by western blotting whole gWAT tissue (Figures 6E and S4).

### 3.7 HuR Inhibition Improves Adipose Anabolism After Fasting in PDAC Mice

**FIGURE 7:**
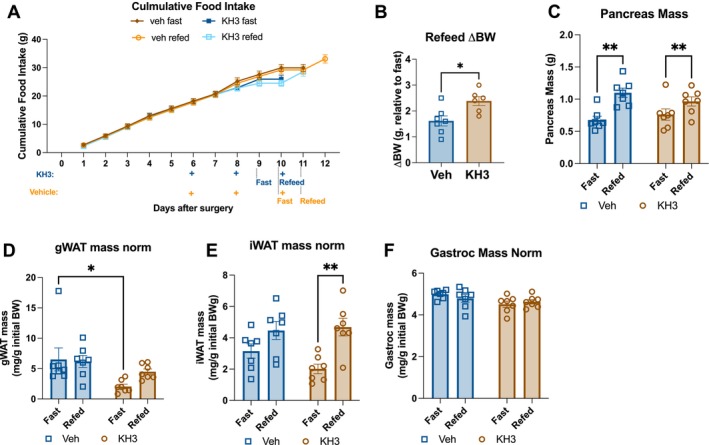
HuR inhibition improves adipose anabolism after fasting in PDAC mice. Wild‐type C57BL/6J mice with PDAC were fed *ad libitum* for 9 days and then fasted 24 h with or without a 24‐h refeed. In each feeding paradigm, groups were treated with either vehicle or the HuR antagonist, KH‐3, and then euthanized 10 or 11 days after tumour implantation following the 24‐h fast or 24‐h refeed. Vehicle‐treated mice were pair‐fed to the KH‐3‐treated group's average food intake from 8 days post‐implantation until study end. *N* = 7 male mice per group. (A) Cumulative food intake, statistically tested as an unpaired *t*‐test of vehicle versus KH‐3 treatment at Day 9 (prior to fast) *p* = 0.1121. (B) Change in body weight after refeeding, statistically tested with unpaired *t*‐test. (C) Terminal pancreas mass. (D) Terminal gWAT mass. (E) Terminal iWAT mass. (F) Terminal gastrocnemius mass. 2 × 2 analyses were statistically tested with two‐way ANOVA with Tukey multiple comparisons. *p < 0.05, **p < 0.01.

**FIGURE 8:**
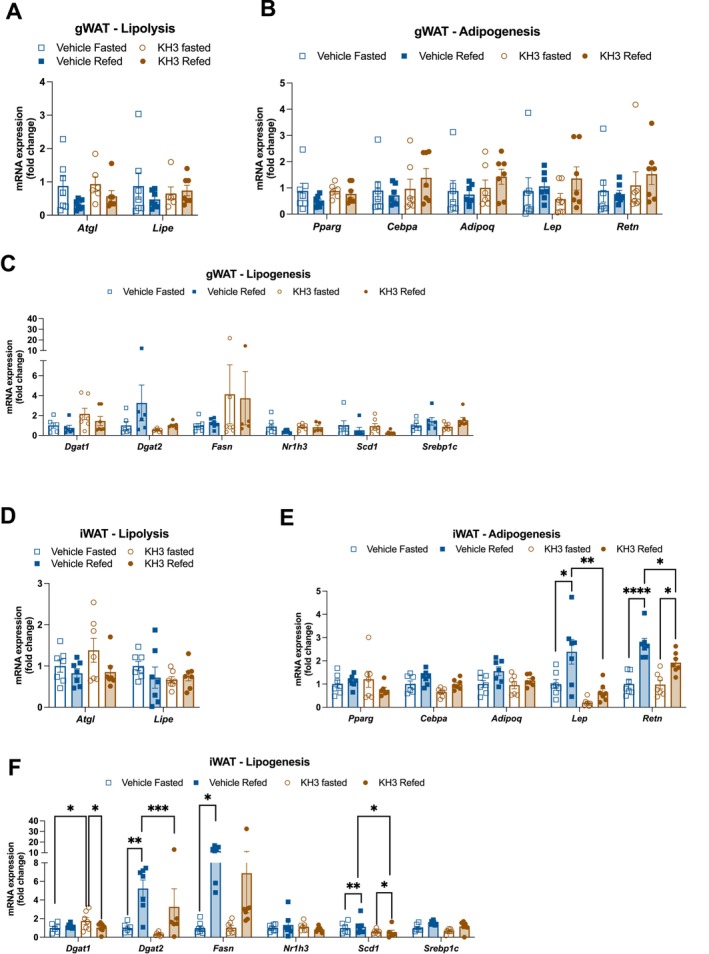
HuR inhibition does not improve gene expression patterns associated with anabolism. mRNA levels of lipolysis (A), adipogenesis (B) and lipogenesis (C) genes in gWAT, normalized to beta‐actin expression. mRNA levels of lipolysis (D), adipogenesis (E) and lipogenesis (F) genes in iWAT, normalized to beta‐actin expression. Data were statistically tested with two‐way ANOVA with Tukey multiple comparisons correction. *N* = 7 male mice per group. *p < 0.05, **p < 0.01, ***p < 0.001, ****p < 0.0001.

Because HuR has been established to drive pro‐survival pathways in PDAC, we next wanted to determine if preventing HuR binding to target mRNAs could reverse adipose wasting in PDAC cachexia [26]. To do this, we treated PDAC mice with a selective HuR inhibitor, KH‐3, which prevents binding of HuR to its target mRNA sequence [33]. All mice received orthotopic PDAC tumour injections and were fed *ad libitum* then fasted 24 h with or without a 24‐h refeed at mid‐cachexia (9 days after injection). In each feeding group, mice were treated with the HuR inhibitor KH‐3 (100 mg/kg) or vehicle at 6‐, 8‐ and 10‐day post‐injection. Cumulative food intake between vehicle and KH‐3 groups was not statistically significant prior to fast. KH‐3‐treated PDAC mice gained significantly more weight during refeeding compared to vehicle‐treated mice (Figure 7A,B). Refeeding was associated with larger tumours in both vehicle and KH‐3 groups, likely due to both log growth and the propensity for mice to become dehydrated during fasting (Figure 7C). KH‐3 treatment itself had no effect on tumour growth over this time interval. In the presence of KH‐3, refeeding increased WAT mass of PDAC animals, which was not observed in vehicle‐treated PDAC mice (Figure 7D–F). These data indicate that mice treated with KH‐3 show signs of functional anabolism that are lacking in PDAC mice given vehicle treatment. This effect is evident despite the fact that KH‐3‐treated mice tended to lose more WAT mass during fasting than vehicle‐treated mice. Pro‐anabolic effects of KH‐3 are specific to adipose tissue, as gastrocnemius muscle mass was not changed between fast/refeed or vehicle/KH‐3 treatment (Figure 7F). KH‐3 treatment did not restore expression of adipogenic (*Cebpa*, *Adipoq*, *Lep* and *Retn*) or lipogenic (*Fasn*, *Dgat1*, *Dgat2*, *Nr1h3*, *Scd1* and *Srebp1c*) genes (Figure 8B–C,E–F). HuR inhibition also did not impact lipolysis gene expression (*Atgl* and *Lipe*) (Figure 8A,D). Thus, we show a role for HuR in adipose tissue metabolism during PDAC‐associated cachexia, whereby inhibiting HuR improved anabolism and adipose retention, although without restoring the expression of genes associated with adipogenesis and lipogenesis.

## 4 Discussion

This work evaluates the relative contributions of enhanced catabolism and impaired anabolism on fat wasting by investigating adipose tissue response to different nutritional contexts and HuR inhibition in cachectic mice. We demonstrate that our murine model of PDAC cachexia exhibits a near‐complete deficit in adipose tissue anabolism in the context of reduced lipolysis. We further identify HuR as a potential molecular mediator of adipogenesis during pancreatic cancer progression. In cultured adipocytes, we showed that PDAC conditioned media does not induce a strong lipolytic response. Although the sensitivity of the glycerol release assay was limited by the absence of BSA as a free fatty acid acceptor, lipolysis and lipogenesis protein and mRNA expression data were congruent with later findings in vivo. Altogether, this emphasizes that PDAC‐derived factors are sufficient to impair adipose anabolism, which corroborates a growing body of literature that challenges the dogma that fat wasting occurs as a result of only enhanced catabolism [16, 17, 18].

Our study implicates the RBP HuR in the inhibition of adipogenesis and lipogenesis in adipose tissue. HuR can impact the transcriptome through both RNA binding and HuR‐dependent splicing [26]. One documented mechanism of HuR‐mediated adipogenesis regulation is via HuR binding to adipogenesis upstream regulator *Pparg*, which suppresses *Adipoq* protein expression in a non‐transcriptionally dependent manner [34]. In our RNA sequencing, we did not observe increased HuR expression, although others reported increased HuR expression in response to nuclear factor‐kappa B (NF‐κB) signalling [35]. Alternatively, we identified HuR as an upstream regulator based on adipose transcriptomic changes associated with PDAC. In this context, HuR is more likely controlled post‐transcriptionally, although further investigation is needed to identify the specific PDAC‐associated factors that may activate HuR stability and nuclear translocation [36].

Small molecule antagonists of HuR, such as KH‐3, are currently in development and have been found to have anti‐tumour effects [26, 37]. In our studies, KH‐3 restored adipose anabolism but also induced severe haemolytic anaemia in all treated mice (Figure S9). Tumour size was not impacted by KH‐3 treatment, likely because of the short period of dosing. However, studies aimed to evaluate tumour growth over 5 weeks documented slower tumour growth and fewer metastases in KH‐3‐treated mice [38]. KH‐3 did not reverse the transcriptional repression of adipogenesis and lipogenesis observed in WAT from PDAC mice. If KH‐3 restores adipogenesis by blocking HuR binding to RNA, we interpret these data to suggest that HuR acts post‐transcriptionally to suppress adipose anabolism, although we cannot rule out non‐HuR‐mediated (i.e., off‐target) mechanisms of action. In our studies, the anti‐cachectic effects of KH‐3 were specific to adipose tissue and did not preserve skeletal muscle mass. Although prior studies link muscle and adipose wasting, these models are all characterized by enhanced lipolysis and browning [30]. As evidenced by the adipose‐specific benefits of KH‐3 with concurrent muscle wasting, in our model, adipose and muscle wasting appear to be driven by independent mechanisms. Continued improvement of HuR antagonists could lead to therapeutics that restore anabolic potential in adipose tissue, providing a novel approach to alleviating one symptom of cachexia, while also providing anti‐tumour benefits.

In addition to impaired anabolic potential in adipose tissue, our PDAC model also presents suppressed lipolysis. Suppressed lipolysis could contribute to systemic defects in energy utilization in cancer cachexia. Prior work from our group shows that PDAC impairs hepatic lipid oxidation but that this does not result in lipid accumulation or fatty liver [32]. It is possible that impaired lipolysis contributes to global metabolic disruption by limiting circulating lipid substrates relative to the degree of caloric deficit. Further interrogation is needed to understand how improved fat retention through HuR inhibition might impact lipid mobilization and systemic metabolism.

Our work identifies impaired adipose anabolism and increased HuR signalling as unique components of adipose wasting in cachexia. Although much of the focus in cachexia research and treatment is on skeletal muscle, multiple recent reports demonstrate that adipose loss in PDAC cachexia is a poor prognostic indicator, independent of muscle loss [5, S9, S10]. This further supports existing work describing heterogeneity within cancer cachexia patients [39]. Moving forward, accounting for cachexia patient heterogeneity, including adipose wasting and loss of anabolic adipose potential, in clinical trial design may lead to increased therapeutic success. The work presented here provides a strong foundation for continuing to evaluate therapeutics designed to improve adipogenesis and lipogenesis in the context of PDAC cachexia.

## Funding

This work was supported by the National Cancer Institute grants K99CA286709 (P.C.A.W.), R37CA280692 (A.J.G.), R01264133 (A.J.G.), K08245188 (A.J.G), R01 CA212600 (J.R.B.), U01CA224012‐03 (J.R.B.) and R21 CA263996 (J.R.B); AACR Grant 15‐90‐25‐BROD (J.R.B.); and the Hirshberg Foundation for Pancreatic Cancer Research (J.R.B.) and support from the Brenden Colson Center for Pancreatic Care (A.J.G., J.R.B. and P.C.A.W.). This work is also supported by the Histopathology Shared Resource for pathology studies (University Shared Resource Program at Oregon Health and Sciences University and the Knight Cancer Institute (P30 CA069533 and P30 CA069533 13S5)). The research reported in this publication used computational infrastructure supported by the Office of Research Infrastructure Programs, Office of the Director, of the National Institutes of Health (S10OD034224). The content is solely the responsibility of the authors and does not necessarily represent the official views of the National Institutes of Health.

## Ethics Statement

The authors of this manuscript certify that they comply with the ethical guidelines for authorship and publishing in the *Journal of Cachexia, Sarcopenia and Muscle* [40]. All human and animal studies were approved by the appropriate ethics committees and were therefore performed in accordance with the ethical standards laid down in the 1964 Declaration of Helsinki and its later amendments. All human subjects provided informed consent, and any identifying information of individual patients has been omitted.

## Conflicts of Interest

The authors declare no conflicts of interest.

## Supporting information


**Figure S1:** Impact of KPC conditioned media on lipolysis in adipocytes. (A) Media glycerol levels, not normalized. Conditioned media and control media alone did not contain glycerol above background (assay buffer only) levels. (B,C) mRNA levels of *Atgl* and *Lipe*. Cells collected 1 and 3 h after KPC CM or isoproterenol (10 μM) treatment. (D,E) Western blot analysis of total HSL and ATGL protein. *N* = 3 wells per condition. Statistically tested with two‐way ANOVA with Tukey correction for multiple comparisons. *p* < 0.05, **p < 0.01, ***p < 0.001, ****p < 0.0001.
**Figure S2:** 3T3‐L1 protein western blot full gels. (A) ATGL and vinculin. (B) HSL and vinculin. (C) p‐HSL S563 and vinculin. (D) p‐HSL S660 and vinculin. Cells collected 1 and 3 h after KPC CM or isoproterenol (10 μM) treatment. *N* = 3 wells per condition.
**Figure S3:** Impact of fasting on lean mass in pancreatic ductal adenocarcinoma‐bearing mice. Wild‐type C57BL/6J mice with PDAC or sham implantations were fed *ad libitum* or fasted 16 h at mid‐cachexia (9 days after injection). Animals were euthanized 10 or 11 days after tumour implantation following the 16‐h fast. *N* = 5 sham, 6 PDAC male mice per feeding condition. (A,B) Body composition changes in fat‐free mass (water subtracted) were characterized at baseline to pre‐fast between sham and PDAC mice (A) and at pre‐ to post‐fast (B). (C) Terminal gastrocnemius muscle mass. 2 × 2 analyses were statistically tested with two‐way ANOVA or mixed effects model with Tukey multiple comparisons. *p < 0.05, **p < 0.01, ****p < 0.0001.
**Figure S4:** gWAT protein western blot full gels. (A) HuR and vinculin. (B) ATGL and vinculin. (C) HSL and vinculin. (D) p‐HSL S563 and vinculin. (E) p‐HSL S660 and vinculin.
**Figure S5:** Unbiased clustering of gWAT RNAseq samples. (A) Principal component analysis of PDAC and sham iWAT samples. (B) Unbiased hierarchical clustering of the top differentially expressed genes.
**Figure S6:** Pancreas mass (containing tumour) from sham and PDAC mice after 24‐h fast, or 24 h fast followed by 24‐h refeeding at 9 days post implantation. 2 × 2 analyses were statistically tested with two‐way ANOVA or mixed effects model with Tukey multiple comparisons. *p < 0.05, **p < 0.01, ****p < 0.0001.
**Figure S7:** Additional images of HuR staining. (A) Pancreas tissue. (B) Gastrocnemius muscle tissue. Scale bars represent 100 μm.
**Figure S8:** Additional images of HuR staining. (A) gWAT tissue. (B) iWAT tissue. Scale bars represent 100 μm unless noted to be 50 μm.
**Figure S9:** Evidence of anaemia in the centrifuged blood from PDAC, KH3‐treated, refed mice. EDTA‐treated blood samples after centrifugation at 2000 g × 20 min to collect plasma. The red blood cell volume (haematocrit) is visibly low after centrifugation, although we did not specifically measure the Hgb or Hct of this blood.
